# Intervention of AXL in EGFR Signaling via Phosphorylation and Stabilization of MIG6 in Non-Small Cell Lung Cancer

**DOI:** 10.3390/ijms241914879

**Published:** 2023-10-04

**Authors:** Ya-Yu Yang, Sheng-Chieh Lin, Jong-Ding Lay, Chun-Yu Cho, Te-Hsuan Jang, Hsiu-Ying Ku, Chih-Jung Yao, Shuang-En Chuang

**Affiliations:** 1 National Institute of Cancer Research, National Health Research Institutes, 35 Keyan Road, Zhunan, Miaoli 35053, Taiwan; irelandfish@nhri.edu.tw (Y.-Y.Y.); jaysclin@gmail.com (S.-C.L.); chunyu0116@nhri.edu.tw (C.-Y.C.); jang@nhri.edu.tw (T.-H.J.); shiuo@nhri.edu.tw (H.-Y.K.); 2 Department of Nursing, National Taichung University of Science and Technology, Taichung 40343, Taiwan; jdlay@nutc.edu.tw; 3 Institute of Molecular Medicine, College of Life Science, National Tsing Hua University, Hsinchu 30013, Taiwan; 4 Department of Internal Medicine, School of Medicine, College of Medicine, Taipei Medical University, Taipei 11031, Taiwan; yao0928@tmu.edu.tw; 5 Cancer Center, Wan Fang Hospital, Taipei Medical University, Taipei 11696, Taiwan

**Keywords:** AXL, MIG6, ERRFI1, EGFR, NSCLC

## Abstract

About 80% of lung cancer patients are diagnosed with non–small cell lung cancer (NSCLC). EGFR mutation and overexpression are common in NSCLC, thus making EGFR signaling a key target for therapy. While EGFR kinase inhibitors (EGFR–TKIs) are widely used and efficacious in treatment, increases in resistance and tumor recurrence with alternative survival pathway activation, such as that of AXL and MET, occur frequently. AXL is one of the EMT (epithelial–mesenchymal transition) signature genes, and EMT morphological changes are also responsible for EGFR–TKI resistance. MIG6 is a negative regulator of ERBB signaling and has been reported to be positively correlated with EGFR–TKI resistance, and downregulation of MIG6 by miR–200 enhances EMT transition. While MIG6 and AXL are both correlated with EMT and EGFR signaling pathways, how AXL, MIG6 and EGFR interplay in lung cancer remains elusive. Correlations between AXL and MIG6 expression were analyzed using Oncomine or the CCLE. A luciferase reporter assay was used for determining MIG6 promoter activity. Ectopic overexpression, RNA interference, Western blot analysis, qRT–PCR, a proximity ligation assay and a coimmunoprecipitation assay were performed to analyze the effects of certain gene expressions on protein–protein interaction and to explore the underlying mechanisms. An in vitro kinase assay and LC–MS/MS were utilized to determine the phosphorylation sites of AXL. In this study, we demonstrate that MIG6 is a novel substrate of AXL and is stabilized upon phosphorylation at Y310 and Y394/395 by AXL. This study reveals a connection between MIG6 and AXL in lung cancer. AXL phosphorylates and stabilizes MIG6 protein, and in this way EGFR signaling may be modulated. This study may provide new insights into the EGFR regulatory network and may help to advance cancer treatment.

## 1. Introduction

Lung cancer is the leading cause of cancer–related deaths worldwide. About 80% to 85% of lung cancer patients are diagnosed with non–small cell lung cancer (NSCLC). NSCLC is mainly divided into three subtypes: adenocarcinoma, squamous cell carcinoma, and large cell carcinoma. Although originating from different types of lung cells, their treatment and prognosis are often similar. More than 90% of the known activating EGFR mutations are the deletion of exon 19 (in–frame) and the point mutation of exon 21 (L858R); thus, EGFR–TKIs have become the most popular drugs for treatment. However, NSCLC patients cannot avoid developing resistance to EGFR–TKIs [1].

AXL is a receptor tyrosine kinase, a member of the TAM family that also includes TYRO3 and MER. Growth arrest–specific protein 6 (GAS6) serves as a ligand for AXL. GAS6–AXL signaling is crucial for cancer progression and metastasis in several types of cancers [2,3,4] AXL can be activated in both GAS6–dependent and –independent manners [5]. AXL can also be transactivated by HER2 and is required for the metastatic cascade during HER2–positive breast cancer progression [6]. Epithelial–mesenchymal transition (EMT) and AXL overexpression are often observed in EGFR–TKI–resistant lung cancers [7,8,9,10,11]. AXL could be transactivated by EGFR in cetuximab–resistant cells [12] and may serve as a potent target in advanced cancer therapy [8,9,12,13,14]. While EGFR signaling favors proliferation and AXL signaling promotes invasion, Vouri et al. proposed that EGFR–AXL hetero–interaction leads to cancer invasion and progression [15].

Mitogen–inducible gene 6 (MIG6/RALT/gene33/Errfi1) is a cytoplasmic adapter protein and binds EGFR with its C–terminal portion [16,17]. MIG6 is a negative regulator of ERBB signaling [18,19,20,21]. MIG6 phosphorylation by activated EGFR at Y394 increases MIG6’s potency for EGFR inhibition. Prior phosphorylation of MIG6 at Y395 by Src dramatically accelerates Y394 phosphorylation by EGFR. Y394/Y395–phosphorylated MIG6 binds to both wildtype and mutant EGFR and decreases EGFR activity [22]. MIG6 is induced by hypoxia and causes primary tumor dormancy [23]. MIG6 expression is correlated with EGFR–TKI resistance [24,25,26,27], and high expression of MIG6 is associated with EMT and poor prognosis in lung cancer [27]. It can be phosphorylated at S251 by Chk1 upon EGFR activation by EGF, and phosphorylation at S251 attenuates the inhibitory activity of MIG6 against EGFR [28]. MIG6 is also highly phosphorylated at S256 in EGFR–mutated NSCLC to prevent EGFR ubiquitination [29]. DNAJB1 (DnaJ homolog subfamily B member I) is another MIG6–interacting protein which negatively regulates MIG6 by enhancing ubiquitin–dependent proteasomal degradation of MIG6 and positively affects the EGFR signaling pathway [30].

Post–translational modifications in MIG6 are pivotal in EGFR signal regulation. In this study, we report that MIG6 is a novel substrate of AXL and can be phosphorylated at several sites, including Y310 and Y394/395.

## 2. Results

### 2.1. AXL and MIG6 Are Coexpressed in Lung Cancer

**Figure 1:**
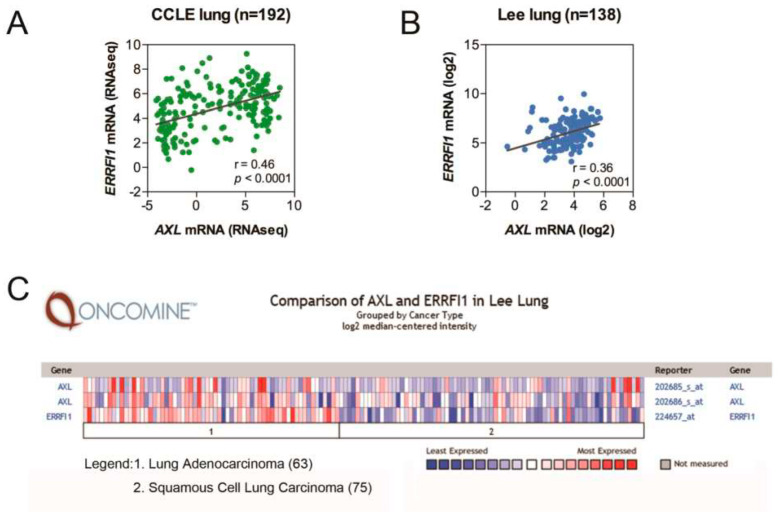
*AXL* and *MIG6* are positively correlated in lung cancer patients. (**A**) A scatter plot generated from lung cancer cell lines (CCLE) showing positive correlation between *MIG6* (*ERRFI1*) and *AXL* mRNA expression levels. (**B**) A scatter plot generated from human primary non–small cell lung cancer (Lee lung) depicting positive correlation between *MIG6* (*ERRFI1*) and *AXL* levels. (**C**) A heatmap generated from primary lung tumors (Lee lung) showing the expression levels of *AXL* and *MIG6* in adenocarcinoma (denoted as 1) and squamous cell carcinoma (denoted as 2).

To assess *AXL* and *MIG6* (*ERRFI1*) mRNA expression in NSCLC patients, we analyzed the Lee lung dataset from Oncomine and the lung cancer cell lines from the CCLE (Cancer Cell Line Encyclopedia). The Wooster cell line dataset from Oncomine, which consists of 298 cell lines from 19 cancer types, was used for analyzing the correlation of the two genes. We found that *AXL* and *MIG6* were coexpressed in many cancers, including pancreatic cancer, prostate cancer and sarcoma (Supplementary Figure S1). Further, we analyzed the correlation of these two genes in the CCLE (Figure 1A) and the Oncomine Lee lung datasets (Figure 1B). The results showed that *AXL* and *MIG6* expression were positively correlated in both the CCLE (Figure 1A) and the Lee lung datasets (Figure 1B) (*p* < 0.0001) and they both had higher expression levels in adenocarcinoma than in squamous carcinoma (Figure 1C).

### 2.2. AXL Overexpression Elevates MIG6 Expression but Downregulates EGFR

**Figure 2:**
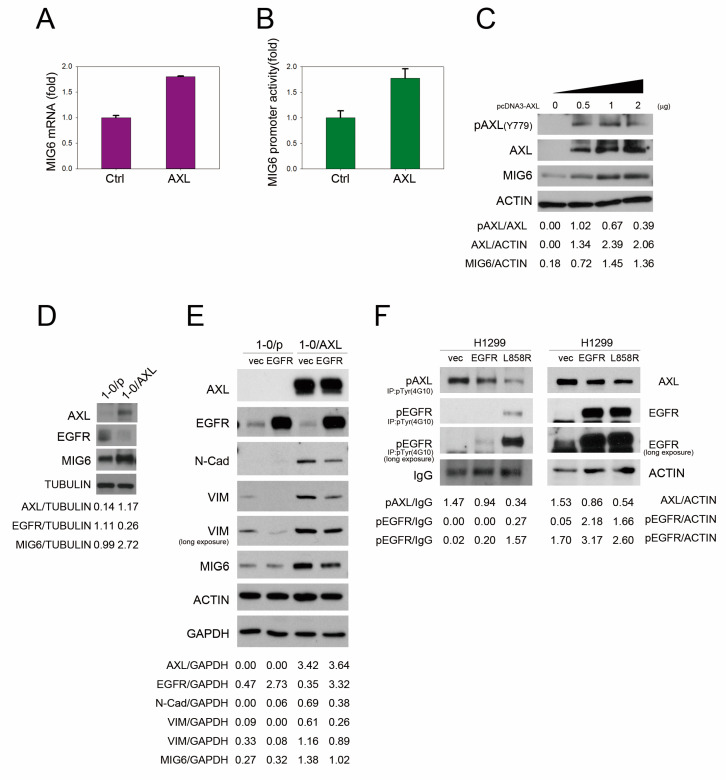
AXL elevates MIG6 and inversely correlates with EGFR activity. (**A**) *MIG6* mRNA expression in CL1–0/pcDNA3 and CL1–0/AXL stable lines assayed with qPCR. (**B**) Reporter assay showing the *MIG6* promoter activity in 293TN cells transfected with or without pcDNA3–AXL plasmid. (**C**) Western blot analysis of pAXL (Y779), AXL and MIG6 in CL1–0 cells. (**D**) Western blot analysis of AXL, EGFR and MIG6 expression in CL1–0/pcDNA3 and CL1–0/AXL stable lines. (**E**) Western blot analysis of AXL, EGFR, N–cad, vimentin and MIG6 expression in EGFR overexpressed CL1–0/pcDNA3 and CL1–0/AXL stable lines. (**F**) Phospho–AXL, AXL, phospho–EGFR and EGFR expression in H1299–vec, H1299–EGFR and EGFR–L858R mutant stable cell lines.

According to our previous study, AXL overexpression in lung cancer cell lines results in elevations in cell invasiveness and drug resistance [31,32]. In this study, by means of an mRNA array, we found that *MIG6* was upregulated by overexpressing AXL in CL1–0 cells. To confirm this finding, quantitative RT–PCR and a promoter reporter assay were performed. As a result, it was found that MIG6 mRNA expression was increased when AXL was overexpressed in CL1–0 cells (Figure 2A). Further, the MIG6 promoter activity was enhanced in AXL–overexpressing CL1–0 cells (Figure 2B). MIG6 expression was also upregulated by ectopically overexpressing AXL in a dose–dependent manner (Figure 2C). Similar results were obtained using the NSCLC cell line PE089 (Supplementary Figure S2). In the CL1–0/AXL stable line, expression of MIG6 and EGFR was up– and downregulated, respectively (Figure 2D). Since AXL drives EMT signaling, we examined EMT traits in the CL1–0/AXL stable line. Indeed, Western blot results showed that mesenchymal markers such as N–cadherin and vimentin were increased in AXL–overexpressing CL1–0 cells (Figure 2E), and importantly, these AXL–increased EMT markers could be significantly reversed by overexpressing EGFR (Figure 2E). When we ectopically expressed either AXL or EGFR, we consistently found that expression of these two genes was inversely correlated. Cell lysates of H1299 overexpressing EGFR wildtype or L858R mutation were immunoprecipitated with an anti–pTyr antibody followed by Western blotting with anti–AXL or an anti–EGFR antibody. The results showed that AXL decreased when EGFR was activated (Figure 2F, left). These observations suggest that AXL drives MIG6 expression and downregulates EGFR in lung adenocarcinoma cells, and imply that MIG6 might play a role in the AXL/EGFR signaling switch.

### 2.3. AXL Stabilizes MIG6 Expression

**Figure 3:**
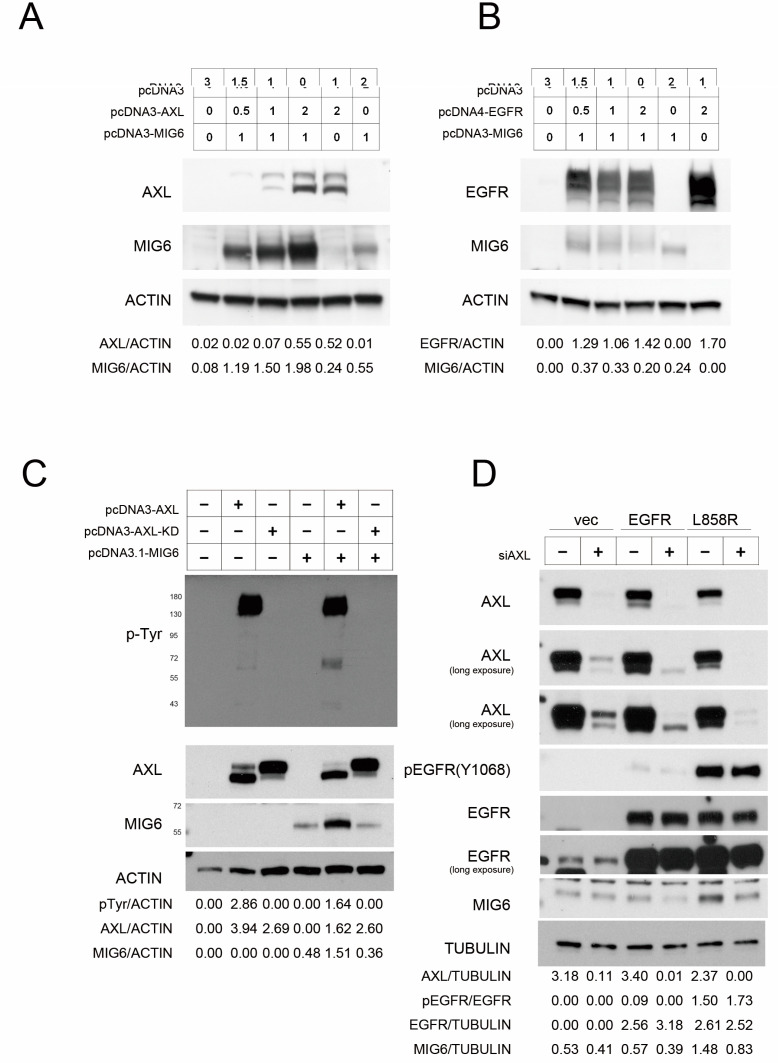
Activated AXL expression stabilizes MIG6 expression. (**A**) Western blot showing the expression levels of AXL and MIG6 among various amounts of pcDNA3–AXL and pcDNA3–MIG6 plasmid–transfected 293TN cells. The plasmid amount that transfected into the cells is indicated in the figure. (**B**) Western blot of anti–EGFR and anti–MIG6 in 293TN cells transfected with different amounts of control pcDNA3, pcDNA4–EGFR and pcDNA3–MIG6 plasmids. (**C**) Western blot of phosphotyrosine (pTyr), AXL and MIG6 levels in MIG6–overexpressing 293TN cells cotransfected with pcDNA3–AXL wildtype (WT) or pcDNA3–AXL–K567R kinase–dead mutant (KD) plasmids. (**D**) Western blot of AXL, phospho–EGFR (Y1068), EGFR and endogenous MIG6 levels in H1299 EGFR wildtype or L858R mutant stable lines with or without anti–AXL siRNA (siAXL). All the lysates were harvested at 72 h after transfection.

We then transfected AXL and MIG6 into 293TN cells to elucidate the relationship of these two proteins. We found that MIG6 was dramatically stabilized in a dose–dependent manner in the presence of AXL (Figure 3A); unlike AXL, EGFR does not exert a stabilizing effect on MIG6 (Figure 3B). We cotransfected MIG6 with wildtype (WT) or kinase–dead (KD; K567R) AXL in 293TN cells. The data showed that only the activated AXL could stabilize MIG6 protein (Figure 3C). We observed that endogenous MIG6 was decreased after AXL was knocked down by siRNA in H1299–EGFR WT or H1299–L858R mutant cells (Figure 3D). These data suggest that AXL is critical for maintaining MIG6 protein stability.

### 2.4. MIG6 Interacts with AXL and Activated AXL Phosphorylates MIG6 on Residues Y310 and Y394/Y395

**Figure 4:**
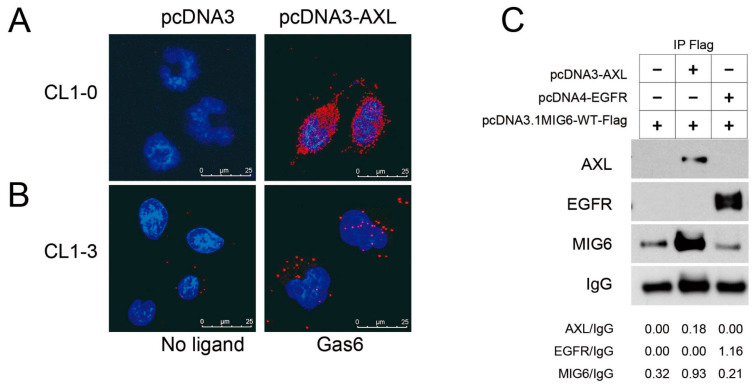
AXL colocalizes and interacts with MIG6. (**A**) PLA assay using anti–AXL and anti–MIG6 antibodies was performed in CL1–0 cells after transfection with pcDNA3 or pcDNA3–AXL for 24 h. (**B**) PLA assay using anti–AXL and anti–MIG6 antibodies was performed in CL1–3 cells treated with or without Gas6 (1 μg/mL) for 15 min. (**C**) Coimmunoprecipitation assay to detect the association of MIG6 with AXL or EGFR. 293TN cells were transfected with pcDNA3, pcDNA3–AXL, pcDNA4–EGFR and/or pcDNA3–MIG6–Flag plasmid. Cell extracts were subjected to coimmunoprecipitation with anti–Flag antibody, followed by Western blotting with the antibodies against AXL, EGFR and MIG6, respectively. Cell lysates were harvested at 72 h after transfection.

**Figure 5:**
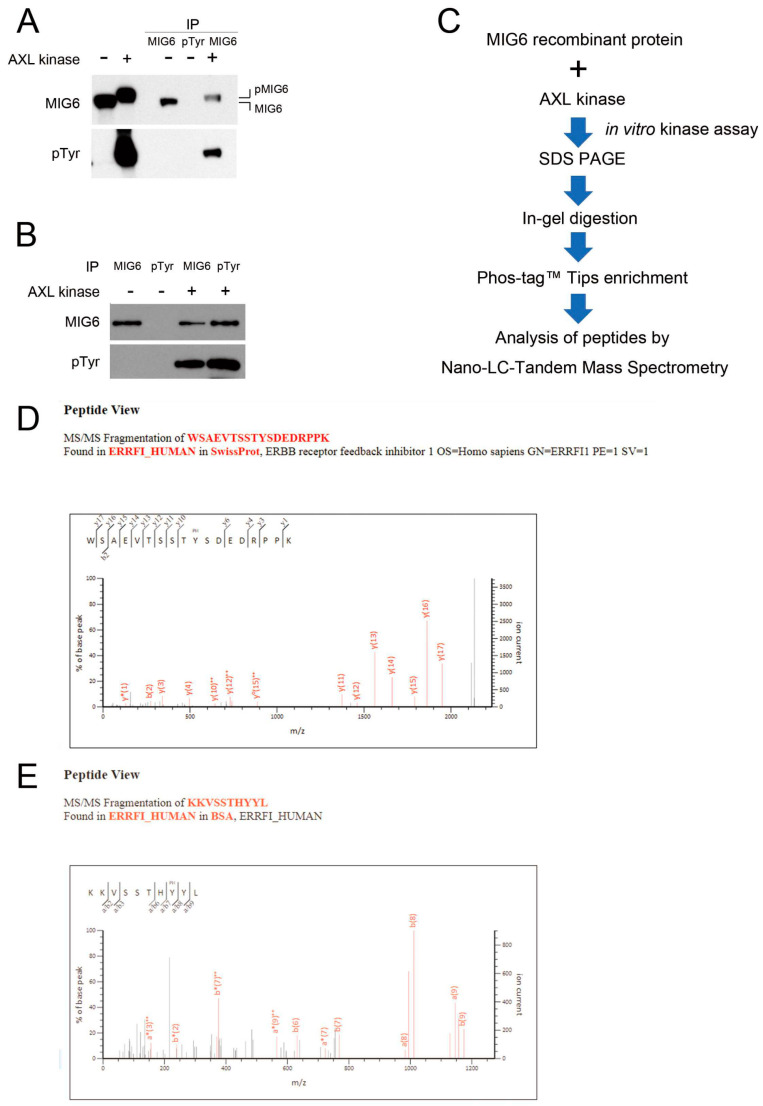
MIG6 is a novel substrate of AXL. (**A**) In vitro kinase assay of AXL on MIG6. Recombinant MIG6 protein was incubated with or without active AXL kinase at 30 °C for 30 min. Incubated proteins were immunoprecipitated with specific antibodies followed by SuperSep™ Phos–tag™ SDS–PAGE and Western blotting against MIG6 and pTyr, respectively. (**B**) In vitro kinase assay of AXL on MIG6. Proteins were separated via SDS–PAGE or Western blotting against MIG6 or pTyr. (**C**) Schematic flowchart for determining the sites of MIG6 phosphorylated by AXL. (**D**) Y310 phosphorylation site of recombinant MIG6 protein determined via LC–MS/MS. (**E**) Y394/Y395 phosphorylation site of recombinant MIG6 protein determined via LC–MS/MS.

**Figure 6:**
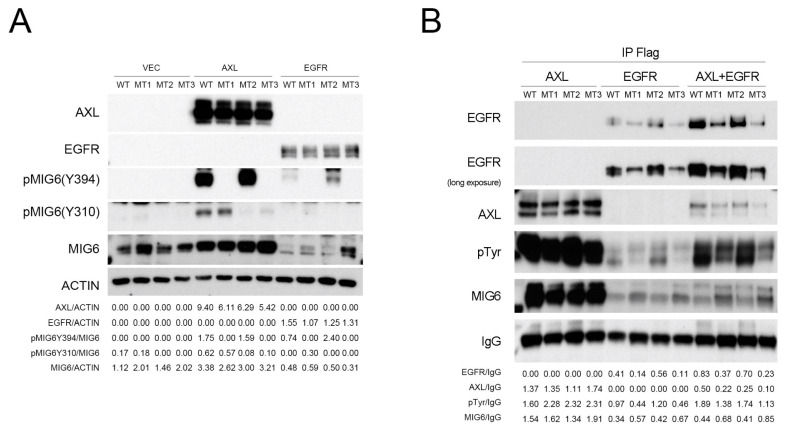
Y310 and Y394/Y395 of MIG6 determine the binding ability of EGFR. (**A**) Immunoblotting (WB) analysis of cell lysates prepared from HEK–293TN cells coexpressing MIG6 variants and AXL or EGFR, with antibodies against phospho–MIG6–Y394, phospho–MIG6–Y310, MIG6, AXL or EGFR. Mammalian expression vectors pcDNA3, pcDNA3–AXL, pcDNA3.1–MIG6WT–Flag (MIG6–WT), pcDNA3.1–MIG6Y394F/Y395F–Flag (MIG6–MT1), pcDNA3.1–MIG6Y310F–Flag (MIG6–MT2), pcDNA3.1–MIG6Y310F/Y394F/Y395F–Flag (MIG6–MT3) and pcDNA4–EGFR were transfected into 293TN cells. Cell lysates were harvested after 72 h. Actin served as the loading control. (**B**) IP with anti–Flag antibody followed by Western blotting with antibodies against, respectively, EGFR, AXL, pTyr and MIG6, as indicated. Anti–IgG served as the loading control.

Because AXL kinase activity is crucial for MIG6 protein stability, we hypothesized that they might interact with each other. We utilized a proximity ligation assay (PLA) to identify protein–protein interactions in lung adenocarcinoma cells. We identified interactions between AXL and MIG6 in situ in CL1–0 cells ectopically expressing AXL (Figure 4A). To mimic and verify whether ligand–induced activated AXL interacts with MIG6 in a tumor microenvironment, we applied GAS6, a ligand for the AXL receptor, to activate AXL in vitro. The red immunofluorescence PLA signals showed protein–protein interactions between AXL and MIG6 upon ligand induction in AXL^high^ CL1–3 cells (Figure 4B). IP Western results also showed that both AXL and EGFR were coimmunoprecipitated with MIG6 when ectopically coexpressed with pcDNA3–MIG6–Flag in 293TN cells (Figure 4C). Labots et al. evaluated a tyrosine kinase peptide microarray for tyrosine kinase inhibitor therapy selection. Their data showed that AXL and Src share most of their peptide substrates; only a few peptides are specific for Src or AXL [33]. Recent studies showed that EGFR can phosphorylate MIG6 at Y394/395 and Src can phosphorylate MIG6 at Y395 [22]. Thus, we wondered if MIG6 could be one common substrate for Src, EGFR and AXL. Since we proved that AXL interacts with MIG6, we then employed an in vitro kinase assay with recombinant MIG6 protein to determine if MIG6 can be phosphorylated by AXL. SuperSep™ Phos–tag™ PAGE was applied to examine MIG6 phosphorylation status. As a result, we saw a MIG6 band shift after incubation with AXL kinase (Figure 5A). IP Western analysis also confirmed that MIG6 was phosphorylated by AXL, i.e., MIG6 was a substrate of AXL (Figure 5B). We then prepared samples for LC–MS/MS analysis to determine the phosphorylation sites (Figure 5C). By means of phosphopeptide enrichment with Phos–tag™ Tips, we saw two peptides with phosphotyrosine. The first peptide showed a solid signal of phosphorylation at the Y310 site of MIG6 (Figure 5D). The second peptide showed a high probability of phosphorylation at Y394 or Y395 (Supplementary Figure S3A). Using semiquantitative analysis, we counted a total of 332 identified peptide fragments displaying phosphorylation at Y394 (52.7%) or Y395 (10.8%) or both Y394/395 (36.4%). We then examined the ability of AXL to directly phosphorylate a synthetic peptide spanning potential sites (residues 387–402) and followed this with LC–MS/MS analysis. Residue Y394 showed a 90.79% possibility of phosphorylation, much higher than that of Y395 (Figure 5E; Supplementary Figure S3B). To further investigate the function of these phosphorylation sites, two constructs of MIG6–MT1 (Y394F/395F) and MIG6–MT2 (Y310F) were used. We knew from previous studies that Y394F/395F mutation disrupts EGFR–MIG6 interaction [22]. Our data showed consistency with these previous findings. In our study, at least two sites of MIG6 were phosphorylated by AXL. Y310 is a specific phosphorylation site for AXL (Figure 6A), and this site was the most important site phosphorylated by AXL. AXL still retained a basal affinity to MIG6–MT1 but had a significantly decreased affinity to MT2 (Figure 6B). The Y310F mutation partially decreased MIG6–AXL interaction, but enhanced MIG6–EGFR interaction (Figure 6B). We hypothesize that phosphorylation of Y310 has a negative effect on MIG6–EGFR interaction. When AXL and EGFR were coexpressed, MIG6 binding activity with these two proteins increased. These data indicate that AXL, EGFR and MIG6 may form a complex, and AXL regulates MIG6/EGFR binding through phosphorylation of MIG6.

## 3. Discussion

Activation of AXL represents a significant mechanism of acquired resistance in cancer cells. Our previous studies revealed that AXL not only plays a critical role in promoting cancer cell invasiveness but may also contribute to chemoresistance. Instead of pursuing direct AXL inhibitors, our research is centered on uncovering AXL’s regulatory mechanisms, which could provide insights into mitigating its effects. In the context of EGFR–driven lung tumorigenesis, MIG6, an EGFR–negative regulator, acts as a tumor suppressor by downregulating EGFR signaling, highlighting the emerging research focusing on the interplay between AXL, MIG6 and EGFR in NSCLC. Our study demonstrates a crucial link whereby AXL positively influences MIG6 protein stability by phosphorylating its Y310 and Y394/395 residues, potentially playing a pivotal role in mediating MIG6’s negative regulation on EGFR signaling in NSCLC.

Our analysis of microarray data uncovered an elevated MIG6 expression when AXL was overexpressed in CL1–0 cells, prompting an investigation into the interplay among AXL, MIG6 and EGFR in NSCLC. Notably, this coexpression pattern between AXL and MIG6 was confirmed in cancer cell lines and NSCLC patients, suggesting potential collaboration in regulating EGFR signaling. MIG6 regulation is critically dependent on the phosphorylation of Y394/Y395 [34]. These phosphorylation events, mediated by c–Abl, activate c–Abl through enhanced autophosphorylation or Y412 phosphorylation [34]. Located within the C–terminal ACK1 homology region (AHR) of MIG6, the EBR serves as a pivotal region for signaling and cell fate determination [35]. Under conditions of EGF deprivation, MIG6, via AHR, establishes connections with the catalytic site and the C–lobe of c–Abl in mammary epithelial cells, inducing cell death and influencing mammary duct formation [34]. Intriguingly, MIG6 also functions as an EGF sensor. In the presence of EGF, Y394/Y395 phosphorylation of MIG6 by EGFR and Src initiates a feedback loop, effectively downregulating activated EGFR [22]. These phosphorylation sites engage with various kinases, participating in multiple signaling pathways, unveiling the intricate interplay among these molecules in cancer signaling regulation.

Distinct phosphorylation sites in MIG6 contribute to EGFR signaling regulation, migration, invasion and tumor progression. MIG6 undergoes phosphorylation by various kinases, including Ser/Thr kinases and Tyrosine kinases. While EGFR and Src–mediated phosphorylation at Y394/Y395 downregulates the activity of EGFR [22], S256 and S251 phosphorylation prevents EGFR signaling downregulation by reducing EGFR ubiquitination [28,29]. Our study introduces Y310 as a novel phosphorylation site within MIG6, demonstrating its potential to disrupt EGFR/MIG6 interactions. Positioned between proline–rich regions (PRRs), and known for protein–protein interactions, especially with SH3 domains [36,37], Y310’s importance lies in MIG6’s adapter role with SH3 and SH2 domains. Mutations at Y394/Y395 or Y310 could impact interactions involving MIG6–AXL and/or MIG6–EGFR, influencing diverse aspects of signaling regulation.

In EGFR mutant lung cancers, elevated MIG6 RNA and protein levels were observed. The Y394/Y395 phosphorylation level rises upon EGFR inhibition [22], potentially enhancing interactions between mutant EGFRs and MIG6, stabilizing EGFR levels. Although MIG6’s role in shielding EGF–induced mutant EGFR internalization remains debated, its undisputed function in preserving EGFR stability in lung cancer cells is evident [29,38]. Our findings extend this understanding by showing that MIG6 also safeguards AXL from GAS6–induced internalization, mirroring observations in the EGFR/MIG6 context (Supplementary Figure S4). This highlights MIG6’s crucial role in maintaining AXL stability.

In the context of tumor progression, proliferation and metastasis are pivotal. AXL signaling primarily promotes invasion, while EGFR signaling favors proliferation, and their interaction contributes to cancer metastasis [15]. Our findings align with this perspective, underscoring the critical role of MIG6 in balancing EGFR and AXL signaling. The EMT gene signature, a predictor of resistance to EGFR inhibitors, correlates with MIG6 expression levels and EGFR–TKI resistance [24,37]. Notably, AXL–induced EMT transition is a common occurrence in many TKI–resistant lung cancers, including those resistant to Osimertinib, a third–generation EGFR–TKI that may induce AXL [39]. Furthermore, our research suggests that the presence of AXL enhances the binding between MIG6 and EGFR, as demonstrated in Figure 6B, potentially implicating MIG6 in mediating AXL/EGFR interactions (Figure 2). The frequent co–overexpression of AXL and MIG6 in metastatic prostate cancer, supported by Oncomine data (Supplementary Figure S1), further reinforces our hypothesis.

**Figure 7:**
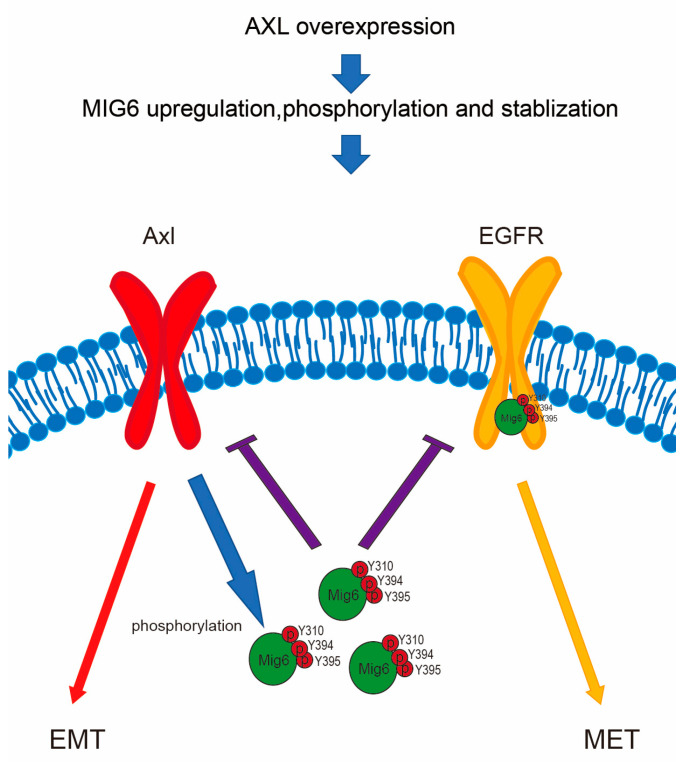
Model for AXL modulating EGFR signaling by phosphorylating and enhancing MIG6 stability in NSCLC. AXL expression induces EMT markers and enhances MIG6 expression. AXL kinase activation leads to phosphorylation of MIG6 at Y394/395 and Y310, resulting in the inhibition of EGFR signaling. Phosphorylated MIG6 subsequently interacts with EGFR, further suppressing EGFR signaling in NSCLC cells.

Our study reveals the intricate interplay between AXL, MIG6 and EGFR signaling in cancer (Figure 7). AXL drives MIG6 expression to downregulate EGFR in lung adenocarcinoma cells, suggesting MIG6’s pivotal role in orchestrating this switch. Furthermore, AXL overexpression impacts not only MIG6 but also PTPN13 (Supplementary Figures S5 and S6), a Her2 regulator, suggesting broader effects on ErbB family genes. These findings can inspire future research focusing on understanding the underlying molecular mechanisms, exploring therapeutic interventions and assessing clinical applications. These insights hold promise for advancing cancer therapy and precision medicine.

## 4. Materials and Methods

### 4.1. Cell Lines

A human lung adenocarcinoma CL1 series of cell lines (CL1–0 and CL1–3) was established by Chu YW et al. via selection of cells in a transwell invasion chamber [40]. Cell lines used in this study were maintained in RPMI–1640 (NSCLC cell lines CL1–0, CL1–3 and H1299; all are EGFR wildtype cells), MEM (NSCLC cell line PE089; EGFR exon 19 deletion) or DMEM (embryonic kidney cell 293TN) supplemented with 10% fetal bovine serum and 2 mM L–glutamine, 100 μg/mL streptomycin and 100 U/mL penicillin, in a humidified 5% CO_2_ atmosphere. CL1–0/pcDNA3 and CL1–0/AXL stable cell lines were established by selecting with G418 (650 μg/mL) as previously described [31]. H1299 EGFR stable lines were established by Dr. YR Chen as previously described [41].

### 4.2. Public Domain Data Mining and Statistical Analysis

In this study, we acquired public gene expression profiling datasets from three different sources: Oncomine, TCGA and the CCLE. We computed associations among the various genes and their levels by employing the Pearson rho correlation coefficient. Significance was established at *p* < 0.05. All statistical analyses were executed utilizing SPSS software, specifically version 22.0, developed by SPSS Inc. in Chicago, IL, USA. Additionally, we performed NLS site prediction using the cNLS Mapper, accessible at http://nls–mapper.iab.keio.ac.jp/ (accessed on 23 November 2020) [34].

### 4.3. mRNA Microarray Analysis

Briefly, total RNA from CL1–0 or AXL–overexpressing CL1–0 was extracted and analyzed using an Agilent Human 1A (V2) Oligo Microarray (Agilent, Santa Clara, CA, USA). The normalized expression ratio was determined using the statistic method “rank consistant lowess”, and the expression ratio was confirmed via RT–PCR. Genes with expression changes greater than 2–fold were selected for further analysis.

### 4.4. Construction of DNA Vectors

Constructs of wildtype (WT) and kinase–dead (KD; K567R) AXL were made as described previously [31,32]. *MIG6* coding region fragments were obtained via RT–PCR from the CL1–3 cell line. PCR primer pair of *MIG6* (NM_018948), namely 5′–GGGGA TCCGC CTCAC AGGTT TGGAG ATG and 5′–GGCTC GAGAC CTCTG CTGAA CCATG ACC, was used for cloning. PCR product of MIG6 fragment was digested by BamHI and XhoI then cloned into pcDNA3 vector. MIG6WT–Flag and MIG6 mutant constructs ((MT1–Y394F/Y395F); (MT2–Y310F); (MT3–Y310F/Y394F/Y395F)) were customized with GenScript (Piscataway, NJ, USA) and were cloned into pcDNA3.1+/C–(K)–DYK.

### 4.5. Reporter Assay

*MIG6* promoter (−2015 to +1 bp) was synthesized and cloned into a pGL3–Basic vector using the XhoI and HindIII sites. 293TN cells (80% confluency) were transfected with the pGL3–MIG6 promoter reporter with or w/o pcDNA3–AXL plasmid, along with pRL–TK Vector (Renilla Luciferase Control Reporter Vectors). Cell extracts were prepared for the luciferase activity assay by using the Dual–Luciferase^®^ Reporter Assay System (Promega Corp., Madison, WI, USA) 48 h post–transfection. Luciferase activity was detected by using an Orion L Microplate Luminometer (Titertek–Berthold, Germany). The ratio of luciferase activity to Renilla luciferase activity was calculated as normalized luciferase activity.

### 4.6. PLA Assay

A proximity ligation assay (PLA) was employed to enable the localized detection of endogenous protein–protein colocalization involving MIG6 and AXL. We procured Duolink^®^ PLA reagents from Sigma–Aldrich, based in St. Louis, MO, USA. For the experimental procedure, CL1–0/pcDNA3, CL1–0/AXL stable cell lines and CL1–3 cells were cultured on glass cover slides. After an overnight incubation, they were subsequently fixed using ice–cold 100% methanol for 15 min at −20 °C. Following fixation, the samples were gently washed three times with PBS, and a blocking solution was applied to them, allowing for a 30 min incubation at 37 °C. Primary antibodies utilized in this study included the MIG6 antibody (sc–66966), sourced from Santa Cruz Biotechnology, and the AXL antibody (ab89224, Abcam, Cambridge, UK). These antibodies were diluted to a ratio of 1:100 in a suitable buffer, and they were added to the samples, which were then subjected to an overnight incubation at 4 °C. Afterward, the samples were washed in PBST (0.01% Tween 20) and subsequently treated with PLA plus and minus probes, and incubated for 60 min at 37 °C. This was followed by two additional PBST washes. The ligation step was performed by applying ligase for 30 min at 37 °C, which was succeeded by an amplification step using polymerase, carried out for 120 min at 37 °C. Subsequently, the samples were washed twice with 2× SSC, followed by a wash with 0.2× SSC. Finally, the samples were mounted using a suitable mounting gel containing DAPI, and signal detection was accomplished through confocal microscopy.

### 4.7. Coimmunoprecipitation (Co–IP) Assay

Cell lysates were harvested 3 days after transfection. Cells were lysed with IP buffer (Tris 50 mM pH7.4; EDTA 5 mM; NaF 50 mM; Na_3_VO_4_ 0.1 mM; Triton 1%). Whole cell lysates obtained via centrifugation were incubated with 2 μg of primary antibody and Protein G Mag Sepharose (GE Healthcare, 28-9670-70; Sigma-Aldrich, St. Louis, MO, USA) overnight at 4 °C. The immunocomplexes were then washed with IP buffer five times and separated via SDS–PAGE. Immunoblotting was performed following standard procedures.

### 4.8. Western Blotting

Briefly, cell lysates were prepared in Triton X–100 lysis buffer (Tris 50 mM pH7.4; EDTA 5 mM; NaF 50 mM (1.047 g/500 mL); Na_3_VO_4_ 0.1 mM; Triton 1%) supplemented with protease and phosphatase inhibitors. Following separation via SDS–PAGE, proteins were transferred onto a PVDF membrane, and nonspecific binding was blocked with 5% skim milk for 1 h. Specific primary antibodies were applied and incubated at 4 °C overnight, and then horseradish peroxidase–conjugated secondary antibodies were added and incubated for an additional 1 h at room temperature. Signal detection was achieved using a chemiluminescence system, and antibodies used in this study are provided in the Supplementary Materials.

### 4.9. In Vitro Kinase Assay and Phosphoprotein Enrichment

Recombinant MIG6 protein 25 μg (H00054206-P01, Abnova, Taipei, Taiwan), active AXL kinase 2.5 μg (A34-11H-10, SignalChem Biotech Inc., Richmond, BC, Canada) and 2 mM ATP in buffer were incubated at 30 °C for 30 min. The reaction was terminated with 50 μL 2X SDS sample buffer. The samples were heated to 100 °C for 5 min, then loaded on SDS–PAGE gel. The samples were analyzed via Western blotting followed by/or in–gel digestion for LC/MS/MS analysis. Phospho–MIG6 peptides were enriched with Phos–tag™ Tip following manual instructions. Phos–tag™ Tip and SuperSep™ Phos–tag™ were purchased from Wako Chemicals (Osaka, Japan). The MIG6 peptide (KKVSSTHYYLLPERPP; 387–402) for in vitro kinase assay was synthesized with GenScript (Piscataway, NJ, USA).

### 4.10. Analysis of Peptides with Nano–LC–Tandem Mass Spectrometry

Peptides, whether they were derived from tryptic digestion or synthesized, underwent separation in which a NanoAcquity UPLC system (Waters, Milford, MA, USA) with a 5 μL sample loop was employed. Mobile phase A contained 0.1% formic acid in H_2_O, while mobile phase B had 0.1% formic acid in 100% methyl cyanide. A 2 μL sample was injected and loaded at a flow rate of 3 μL/min in aqueous 0.1% (*v*/*v*) formic acid via a Symmetry C18 5 μm, 2 cm × 180 μm trap column (Waters) prior to separation. Separation was conducted at 40 °C through a bridged ethyl hybrid C18 column (1.7 μm, 25 cm × 75 μm analytical reversed–phase column).

Peptides were eluted from the column using the following gradient: 3–60% B over 85 min, 60–85% B for 1 min and 85–85% B for 9 min, all at a flow rate of 300 nL/min. This was followed by a 24 min equilibration at the same flow rate, maintaining the column temperature at 40 °C. For MS and MS/MS, a lock mass solution of 100 fmol/μL [Glu1] fibrinopeptide B (Sigma) was continuously delivered at 500 nL/min by the NanoAcquity’s auxiliary pump to the NanoLockSpray source on the mass spectrometer.

The UPLC system interfaced with a SYN–APT G2 Q–TOF tandem mass spectrometer (Waters) operating in positive ion mode with a mass resolution of ~10,000. Calibration involved using sodium iodine over m/z 50 to 2000, and postacquisition calibration involved employing the doubly protonated precursor ion of [Glu1] fibrinopeptide B. Data acquisition was performed using LC–MS/MS with a data–dependent strategy: 1 s MS survey analysis with a 0.02 s interscan delay, followed by five MS/MS cycles. Fragment ions from the five most abundant multiply charged precursor ions (2+, 3+ and 4+) were detected at an integration rate of 1.2 s with a 0.02 s interscan delay. Collision energy ranged from 20 to 45 eV, and dynamic exclusion of precursors was set to 60 s. The reference sprayer was sampled every 10 s for instrument stability.

### 4.11. LC–MS/MS Data Analysis

The LC–MS/MS raw data were converted into pkl files using the ProteinLynx Global Server (PLGS) program version 2.5. These pkl files were then searched against the in–house database using MAS–COT search engine (version 2.5) with the following parameters: peptide mass tolerance, 100 ppm; fragment mass tolerance, 0.2 Da; trypsin cleavage with a maximum of one missed cleavage; fixed modifications, carbamidomethyl on cysteine; variable modification, oxidation on methionine.

## 5. Conclusions

Here we present that AXL positively regulates MIG6 protein stability by phosphorylating its Y310 and Y394/395 residues. MIG6 plays a crucial role in cell signaling in cancer. In this study, we addressed the significance of MIG6 phosphorylation sites in which the cell fate is determined through signaling regulation.

## Data Availability

The datasets used for this study are available from the corresponding author upon request.

## Supplementary Materials

The following supporting information can be downloaded at https://www.mdpi.com/article/10.3390/ijms241914879/s1.
