# Supplementary material for: Intervention of AXL in EGFR Signaling via Phosphorylation and Stabilization of MIG6 in Non-Small Cell Lung Cancer
Source: Int J Mol Sci. 2023 Oct 4;24(19):14879. doi: 10.3390/ijms241914879 (PMC10573631; doi:10.3390/ijms241914879)
Supplement: Supplementary file 1 [file ijms-24-14879-s001.zip › ijms-2628223-supplementary.pdf]

Supplementary information

Intervention of AXL in EGFR Signaling via Phosphorylation and Stabilization of MIG6 in Non-Small Cell Lung Can-cer

Ya-Yu Yang [1,†], Sheng-Chieh Lin [1,†], Jong-Ding Lay [2], Chun-Yu Cho [1], Te-Hsuan Jang [1,3], Hsiu-Ying Ku [1], Chih-Jung Yao [4,5] and Shuang-En Chuang [1,*]

This file includes:

Reagents and antibodies used in this study.

Primers for qRT-PCR used in this study.

Supplementary figures:

Fig. S1. Comparison of AXL and MIG6 expression in Wooster Cell Line.

Fig. S2. AXL overexpression causes MIG6 upregulation in PE089 NSCLC cell line.

Fig. S3. MIG6 phosphorylation sites analysis.

Fig. S4. MIG6 is a negative regulator of AXL.

Fig. S5. AXL and MIG6 RNA expression levels in primary and metastatic tumors of Chandran Prostate dataset.

Fig. S6. Western blot analysis showing AXL and PTPN13 levels in CL1-0 cells ectopically expressing AXL.

Reagents and antibodies

Reagents:

Chemical inhibitors, LY294002, U0126, and Gefitinib were purchased from Cell Signaling (Danvers, MA, USA). Specific siRNAs of *ERRFI1*, *AXL, EGFR* and scrambled control were products of GE Dharmarcon (Lafayette, CO, USA.). Human recombinant Gas6 was purchased from R&D Systems, Inc., (Minneapolis, MN, USA) and human recombinant EGF was purchased from Thermo Fisher Life Technologies (Waltham, MA, USA).

Antibodies:

Primary antibodies of AXL (sc-1096; amino and carboxyl terminals), Mig6 (sc-66966 and sc-137154) were purchased from Santa Cruz Biotechnology (Dallas, TX, USA). HRP-conjugated anti-goat, anti-mouse, and anti-rabbit secondary antibodies were also from Santa Cruz Biotechnology. Anti-phospho-AXL (Y779), anti-phospho-Mig6 (Y310) and anti-phospho-Mig6 (Y394) were made-to-order from Genetex Tec. Polyclonal pY779-specific anti-AXL antibody was raised by immunizing with the peptide-DGL(Yp)ALMSRC (corresponding to AXL residues 776–785). Polyclonal pY394-specific anti-Mig6 antibody was raised by immunizing with the peptide-KVSSTH(Yp)YLLPER (corresponding to Mig6 residues 388–400). Tubulin (T5168) and ANTI-FLAG® M2 (F3165) antibodies were from Sigma-Aldrich. The AXL antibody (8661) and phospho-Tyrosine (8954) were purchased from Cell Signaling Technology (Beverly, MA, USA). Anti-EGFR serum was a kindly gift from Dr. Yi-Ron Chen (Institute of Molecular and Genomic Medicine, Taiwan, NHRI). Active AXL kinase was purchased by Promega #V3901 (Promega Corp. WI, USA), MIG6 recombinant protein was purchased by Abnova (H00054206-P01). Rabbit anti-pAXL (Y779) was developed by immunizing rabbits with specific peptides (H-Asp-Gly-Leu-Tyr(PO3H2)-Ala-Leu-Met-Ser-Arg-Cys-OH) and subsequently purifying the antibody (GeneTex, Miaoli County, Taiwan.)

qRT-PCR primers

qRT-PCR primers for MIG6 fwd: 5’-TTGCTGCTCAGGAGATCAGA-3’ and MIG6 rev: 5’-TTCAGACTGTAGGCCATGGTT-3’.

Supplementary Figures

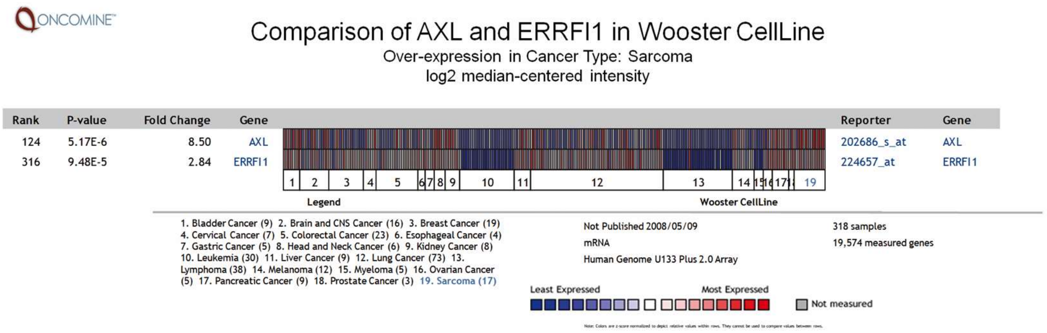

Supplementary Figure S1. Comparison of AXL and MIG6 expression in Wooster Cell Line. Heatmap of AXL and MIG6 transcript levels in various types of cancers including lymphoma, leukemia, myeloma, sarcoma and those of the bladder, brain, breast, cervix, colorectum, esophagus, stomach, head & neck, kidney, liver, lung, pancreas, prostate, and ovarian.

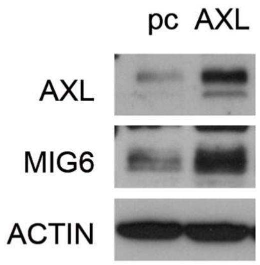

Supplementary Figure S2. AXL overexpression causes MIG6 upregulation in PE089 NSCLC cells. Western blot of AXL and MIG6 expression upon AXL overexpression in PE089 cells.

A

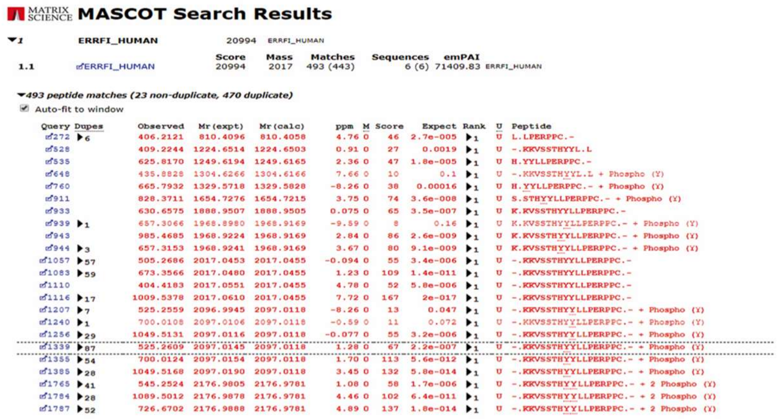

Site	Y394	Y395	Y394 and Y395
Peptides	175	36	121
probability	175/332=52.7%	36/332=10.8%	121/332=36.4%

B

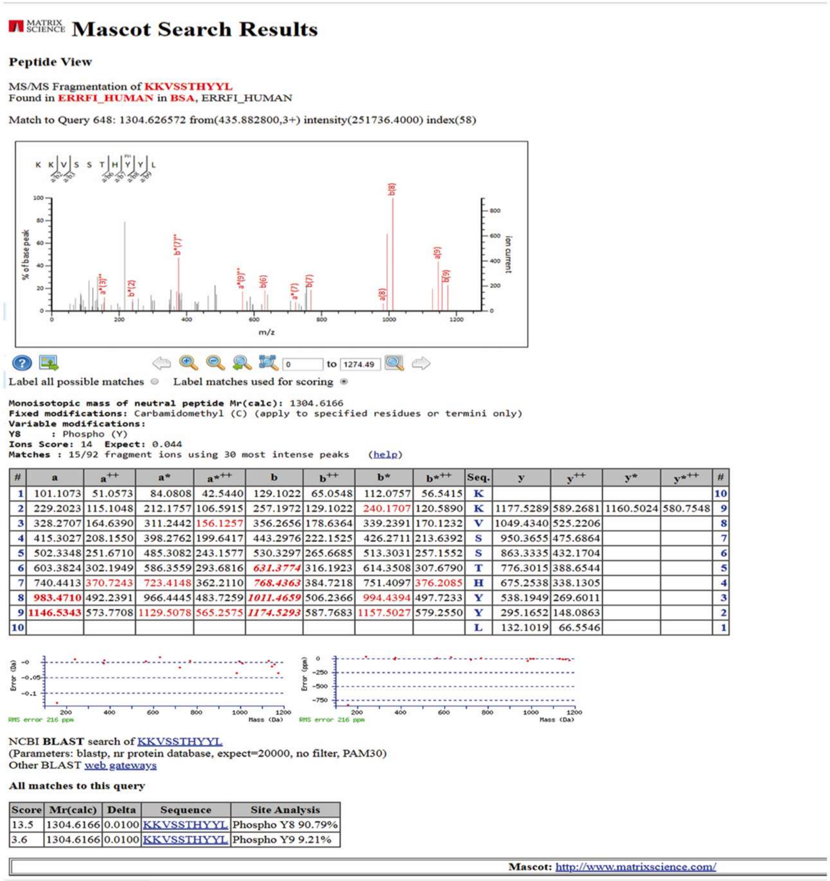

Supplementary Figure S3. MIG6 phosphorylation sites analysis. (**A**) Y394 or Y395 or both Y394/395 are possible sites phosphorylated by AXL. (**B**) A synthetic MIG6 peptide spanning potential sites (residues 387–402) were examined by LC-MS/MS. Y394 showed 90% possibility of phosphorylation.

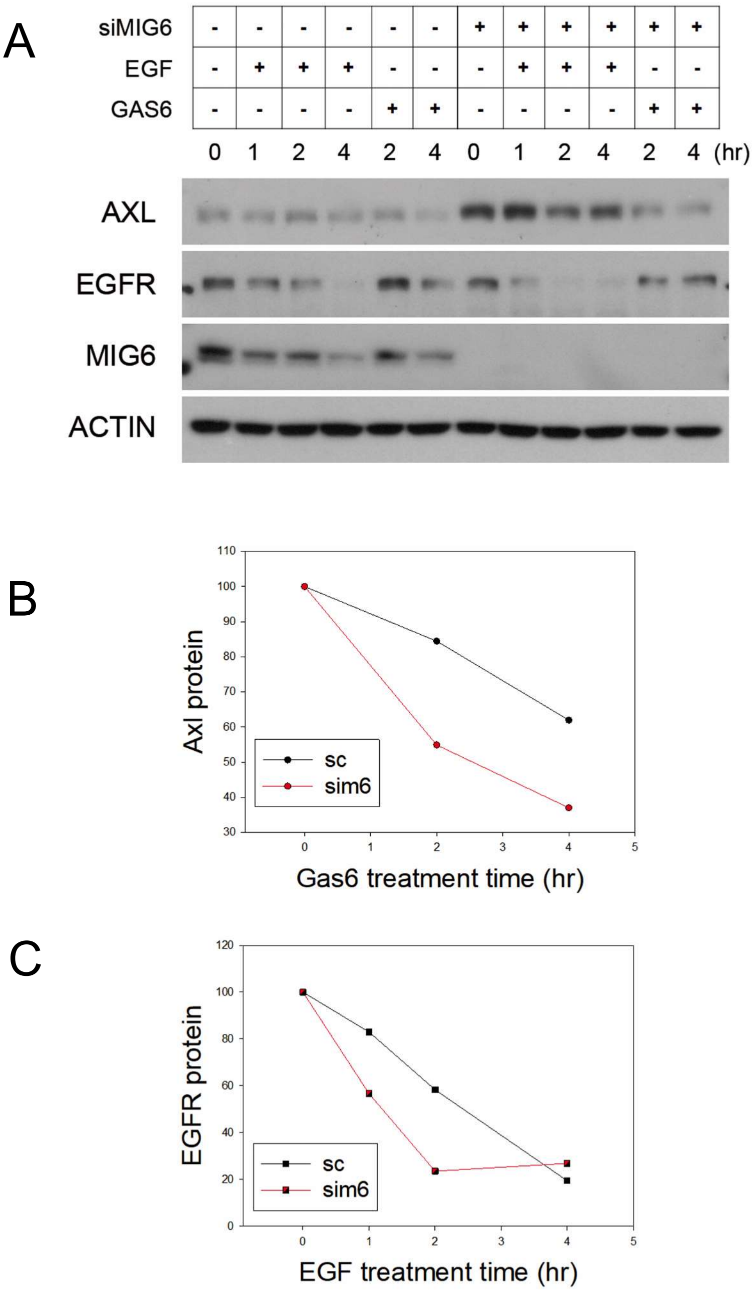

Supplementary Figure S4. MIG6 is a negative regulator of AXL. (**A**) Western blot of AXL and MIG6 levels upon EGF or GAS6 treatment. Cells were transfected with or without anti-MIG6 siRNA (siMIG6). All the lysates were harvested at 72 hrs after transfection. (**B** and **C**) CL1-3 cells were serum-starved for 24 hrs then treated with cyclohexamide for 1 hr followed by treatment with EGF or GAS6 for the time indicated. Quantification plot showed the internalization/degradation rates of AXL (B) and EGFR (C) after treating cells with or without MIG6-silencing.

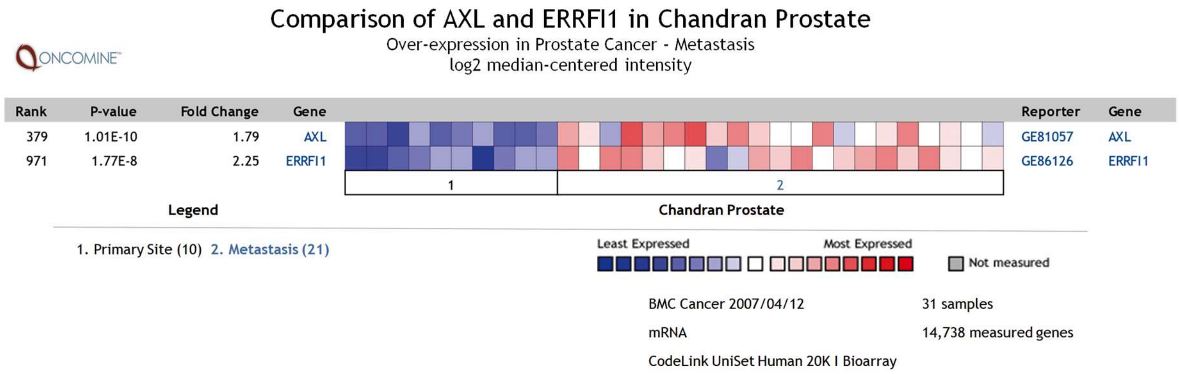

Supplementary Figure S5. AXL and MIG6 RNA expression levels are higher in metastatic tumors comparing with the primary sites. Heatmap shows AXL and MIG6 transcript levels in metastatic and primary tumors of Chandran prostate dataset.

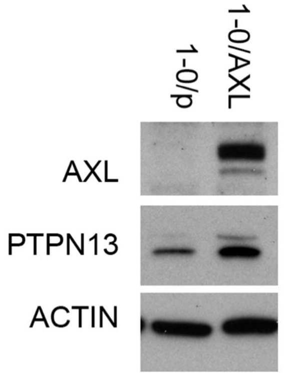

Supplementary Figure S6. AXL overexpression increases PTPN13 expression in CL1-0 cells. Western blot of AXL and PTPN13 expression upon AXL overexpression in CL1-0 cells.
